# Whole-Transcriptome Analysis Reveals Potential CeRNA Regulatory Mechanism in *Takifugu rubripes* against *Cryptocaryon irritans* Infection

**DOI:** 10.3390/biology13100788

**Published:** 2024-10-01

**Authors:** Yuqing Xia, Xiaoqing Yu, Zhen Yuan, Yi Yang, Ying Liu

**Affiliations:** 1 Key Laboratory of Environment Controlled Aquaculture, Dalian Ocean University, Ministry of Education, 52 Heishijiao Street, Dalian 116023, China; xiayuqing_dlou@163.com (Y.X.); y17866561237@163.com (Y.Y.); 2 College of Fisheries and Life Science, Dalian Ocean University, 52 Heishijiao Street, Dalian 116023, China; 3 College of Biosystems Engineering and Food Science, Zhejiang University, 866 Yuhangtang Road, Hangzhou 310058, China

**Keywords:** *Takifugu rubripes*, non-coding RNA, ceRNA, *Cryptocaryon irritans*, immune response

## Abstract

**Simple Summary:**

*Cryptocaryon irritans* is a primitive ciliate that parasitizes the skin of marine fish and can cause illness or even death in Fugu. In this study, we revealed an immune regulatory network of genes and non-coding RNAs co-modulating competing endogenous RNAs. Potential biomarkers of Fugu infection with *Cryptocaryon irritans* were analyzed by bioinformatics. Of these, the regulatory network showed that the LOC105418663-circ_0000361-fre-miR-204a-*fzd3a* ceRNA axis was potentially involved in modulating the immune response of Fugu against *Cryptocaryon irritans* infection. Our research results have laid a theoretical foundation for a deeper understanding of the immune mechanism of Fugu and proposed new treatment and prevention measures for resisting *Cryptocaryon irritans* infection.

**Abstract:**

*Cryptocaryon irritans* (*C. irritans*) is a proto-ciliate parasite that infects marine fishes, including the cultured species *Takifugu rubripes* (*T. rubripes*), causing disease and potential mortality. In host organisms, infection by parasites triggers an immune response that is modulated by regulatory elements including proteins and non-coding RNAs. In this study, the whole transcriptome RNA sequencing of *T. rubripes* gill tissue before and after infection with *C. irritans* was performed to reveal the competitive endogenous RNA (ceRNA) regulatory network. Histomorphology revealed gill segment swelling and parasitic invasion in the infected group. The analysis identified 18 differentially expressed miRNAs (DEMs), 214 lncRNAs (DELs), 2501 genes (DEGs), and 7 circRNAs (DECs) in the infected group. Gene Ontology (GO) enrichment analysis revealed that these genes were notably enriched in the Wnt signaling pathway and mTOR signaling pathway. The co-expression networks (lncRNA/circRNA-miRNA-mRNA) were constructed based on correlation analysis of the differentially expressed RNAs. Further analysis suggested that the LOC105418663-circ_0000361-fru-miR-204a-*fzd3a* ceRNA axis was potentially involved in the regulation of immune responses against *C. irritans* infection. Finally, the expression levels of DEG, DEL, and DEM were validated. This study reveals the regulatory mechanism of a candidate ceRNA network, providing insights into the potential mechanism of *T. rubripes*’ infection with *C. irritans*.

## 1. Introduction

*Takifugu rubripes* (*T. rubripes*) is a well-studied model species of teleost fishes. This coastal fish holds economic significance and can be found in regions including China, Korea, Japan, and other areas [1,2,3]. *T. rubipres* is one of the most economically valuable species [4]. With breakthroughs in breeding technology and the maturation of culture techniques, the cultivation scale of *T. rubripes* has expanded, and production has stabilized in recent years [5]. However, during aquaculture production, fish are susceptible to a variety of infectious diseases that cause significant economic losses [6,7]. Of these, parasitic diseases are the primary diseases in *T. rubripes* aquaculture, posing serious threats to the health of the fish.

*Cryptocaryon irritans* (*C. irritans*) is a type of ciliate protozoan that primarily infects the gills, skin, and fins of its hosts, leading to serious and often deadly infections [1]. As aquaculture continues to expand globally, the threat posed by *C. irritans* in marine fish farming cannot be ignored [1,2]. *C. irritans* can infect almost all marine teleosts with broad host specificity, and high-density mariculture is more likely to lead to large outbreaks of infection and high lethality [2]. After being infected by *C. irritans*, the host produces innate and specific immune responses [8,9], inducing related inflammatory pathways, such as toll-like receptors (TLR) [10]. Currently, research on the control of *C. irritans* is centered on disease prevention, with fewer studies related to disease treatment. Therefore, comprehending the immune response of *T. rubripes* to *C. irritans* infection is crucial for preventing and controlling the disease.

RNA-seq analysis has become a popular method for studying immune mechanisms in recent years. By analyzing expression profiles, researchers can identify immune-related genes that play a critical role in the immune response. This approach allows for a comprehensive understanding of the complex interactions between different genes and their impacts on the immune system [11,12,13]. For example, the cytosolic interleukin IL-34 was significantly up-regulated in the gills of golden pompano (*Trachinotus ovatus*) after stimulation with *C. irritans* infection [14]. There is growing evidence that insights into the dynamics of gene expression regulation can be gained through transcriptional products, including mRNAs and non-coding RNAs (ncRNAs). ncRNA includes microRNA (miRNA), long non-coding RNA (lncRNAs), and circular RNA (circRNA) [15,16]. In contrast, competitive endogenous RNAs (ceRNAs) involve more gene species than the miRNA regulatory network, and form a larger inter-regulatory network of RNA molecules [17,18]. It has been shown that in teleost, the lncRNA MARL can competitively bind miRNA-122 and weaken the targeted inhibition of MAVS by miRNA-122, elucidating the molecular response mechanism of the ceRNA machinery regulating the RIG-I antiviral signaling pathway [13]. In addition to influencing signaling pathways, ceRNAs also participate in the regulation of disease occurrence and development [19,20]. Therefore, association analysis of ceRNAs using transcriptome sequencing is an effective tool for exploring potential regulatory network mechanisms.

In this study, we infected *T. rubripes* with *C. irritans* and analyzed gill tissues for expression differences at the coding RNA and non-coding RNA levels, identifying differentially expressed miRNAs (DEMs), lncRNAs (DELs), circRNAs (DECs), and genes (DEGs). Functional enrichment analysis was then performed to predict key genes involved in immune-related pathways. In addition, we established ceRNA networks including lncRNA-miRNA-mRNA and circRNA-miRNA-mRNA, and performed functional analyses to explore the regulatory network of the immune response in the organism following *C. irritans* infection. The research highlights how the immune system of *T. rubripes* responds to infections and provides insights into its defense strategies. Additionally, it sheds light on the immunological control mechanisms of *C. irritans*. This information is critical for developing new methods of managing infections caused by *C. irritans*, potentially leading to more effective treatments and preventive measures in aquatic environments.

## 2. Materials and Methods

### 2.1. Ethics Statement

The research procedures for all fish were carried out following the Guidelines for Experimental Animals set out by the Ministry of Science and Technology. The Institutional Animal Care and Use Committee at Dalian Ocean University (located in Dalian, China) reviewed and approved the experimental protocols. Anesthesia was induced using MS-222, and efforts were made to minimize pain and discomfort throughout the study.

### 2.2. Experimental Animals

Six healthy individuals of *T. rubripes* (mean length: 9.5 ± 0.9 cm; mean weight: 100 ± 5 g; age: 5 months) were sourced from Dalian Tianzheng Industrial Co., Ltd. (Dalian, China). These experimental fish were randomly distributed into two tanks, each containing three fish, forming three biological replicates: infection group (IG) and control group (CG). Before the experiment, the fish underwent an acclimation period of approximately two weeks in cycling-filtered tanks filled with fresh seawater. Tank conditions were maintained at a volume of 50 L, a temperature of 22.0 ± 0.5 °C, an average salinity of 31, a dissolved oxygen content exceeding 7.0 mg/L, and a pH of 7.5 ± 0.5. The fish density was set at 6 kg/m^3^, 12L:12D. Feeding occurred twice daily at 8:30 and 18:30 h.

### 2.3. Experimental Design

The infection protocol was conducted as previously described [21]. The IG was immersed and exposed to *C. irritans* at a concentration of 1 × 10^4^/fish for three hours. Concurrently, the CG was exposed to the equivalent volume of phosphate-buffered saline (PBS) for an identical period. Subsequently, both groups were transferred to separate clean tanks. Throughout the seven-day experimental period, neither group exhibited any mortality. Anesthesia was induced in all six fish using MS-222, following which gill tissue samples were collected on the seventh day. The aseptic transfer of samples was conducted into sterile containers, followed by flash-freezing in liquid nitrogen, and then storage at a temperature of −80 °C until additional analysis.

### 2.4. Library Construction and Illumina Sequencing

Total RNA was extracted by Trizol, RNA concentration and purity were determined, and, after passing the quality check, the total RNA was sent to Novogene (Beijing, China) to complete sequencing. miRNA-seq, lncRNA-seq, circRNA-seq, and mRNA-seq are described in the Supplementary Materials File S2, which gives details of the methodological steps and differential expression analysis. A total of six libraries were constructed. In miRNA library construction, total RNA was used as the starting sample. Small RNA was constructed for Illumina SE50 sequencing. Bowtie 2.1.1 [22] software was used for small RNA mapping reads. Gene Ontology (GO) and Kyoto Encyclopedia of Genes and Genomes (KEGG) enrichment analyses were implemented using miRanda 2.2.1 [23] and RNAhybrid 2.1.2 [24] for target gene prediction of miRNAs, as well as miRNA quantification and differential expression analysis (Supplementary Table S5).

lncRNA, circRNA, and mRNA libraries were constructed for Illumina PE150 sequencing (Supplementary Table S4). Hisat2 2.1.0 [25] software was used for comparative analysis, and Cuffmerge software 2.0.2 was used to merge transcripts obtained from the splicing of each sample. The circRNAs were identified by find_circ and CIRI 1.1.0 software, and the raw sequences were checked for error rate and comparison rate, and then compared, spliced, screened, quantified, and analyzed for the significance of differences, predicted target gene, and functional enrichment. DEMs, DELs, and DEGs were determined using edgeR 4.0 software and based on padj < 0.05 and |log2(foldchange)| > 1.

### 2.5. Construction of ceRNA Networks

The screened differentially expressed miRNAs, lncRNAs, circRNAs, and mRNAs were analyzed. The target genes of DEMs enriched in the immune pathway were predicted. DEL and DEC, which have targeted relationships and differential expression with target genes and DEM, were predicted separately. The correlation coefficient was calculated simultaneously. Previous studies have shown that lncRNAs and circRNAs act as miRNA sponges to competitively bind to miRNAs, affecting the regulation of the target genes by the miRNAs, and thus modulating the expression of the target genes. The ceRNA network was visualized using Cytoscape (Cytoscape 3.4.0, http://www.cytoscape.org/download.php, accessed on 15 May 2024) and Figdraw (https://www.figdraw.com/static/index.html#/, accessed on 17 June 2024).

### 2.6. Quantitative Real-Time PCR Verification

To further validate the RNA-seq data, 5 DEMs, 5 DELs, and 5 DEGs were randomly identified for qRT-PCR. The total RNA was extracted by TRIzol. RNA was reverse transcribed using a cDNA synthesis kit (Vazyme, China). miRNA first strand cDNA synthesis (Vazyme, China) was performed. Validation of miRNA, lncRNA, and mRNA was conducted with LightCycler 96 using qRT-PCR. U6 served as an internal reference control for miRNA normalization, while *β-actin* was utilized for mRNA and lncRNA. The primer design used Primer 5 and the primer sequences can be found in Supplementary Table S1. Each qRT-PCR assay was independently performed three times, and the 2^−△△Ct^ method was applied for quantification of mRNAs, miRNAs, and lncRNAs.

### 2.7. Statistical Analysis

SPSS 19.0 software (IBM) was used to develop statistics and perform analysis of all data. A Student’s *t*-test was used for the comparison of differences between the two groups. padj < 0.05 was statistically significant.

## 3. Results

### 3.1. Pathological Tissue

**Figure 1:**
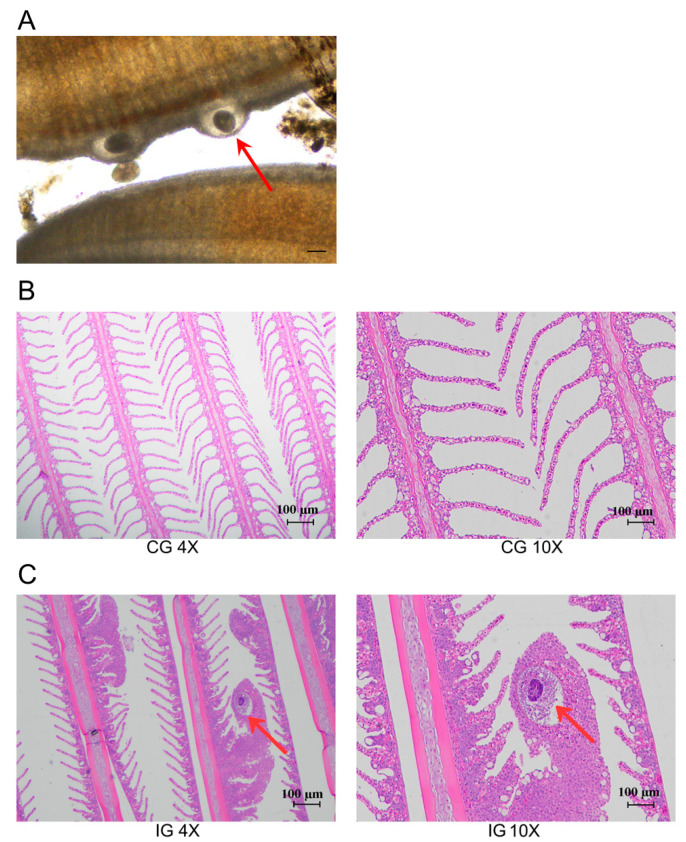
*T. rubripes* infected with *C. irritans*. (**A**) Gill parasitic trophozoite (**B**) HE staining of gills in the CG (**C**) HE staining of gills in the IG. The red arrow points to the trophont of *C. irritans*. The parasitic stage on the *T. rubripes* is called the trophont and is round or pear-shaped, capable of rotational movement within the superficial epithelial layer.

After the infection of the *C. irritans* in the *T. rubripes*, an optical microscope was used to observe the parasitic trophozoites on the gill filaments, which were round or oval in shape (Figure 1A). HE staining was used to further observe the microstructure of the gills in the CG and IG. The findings indicated that the CG exhibited a distinct gill structure, characterized by slender filaments devoid of mucous cells on the gill surface, and an absence of inflammation (Figure 1B). Conversely, the IG displayed gill filaments covered in numerous mucous cells, which had led to swelling and inflammation. The accumulation of mucus obstructed the capillaries within the gills, indicative of hypoxic conditions. At the same time, parasitic trophozoites were observed in the paraffin section of the gill filaments in the IG (Figure 1C).

### 3.2. RNA Sequencing and cDNA Library Construction

**Figure 2:**
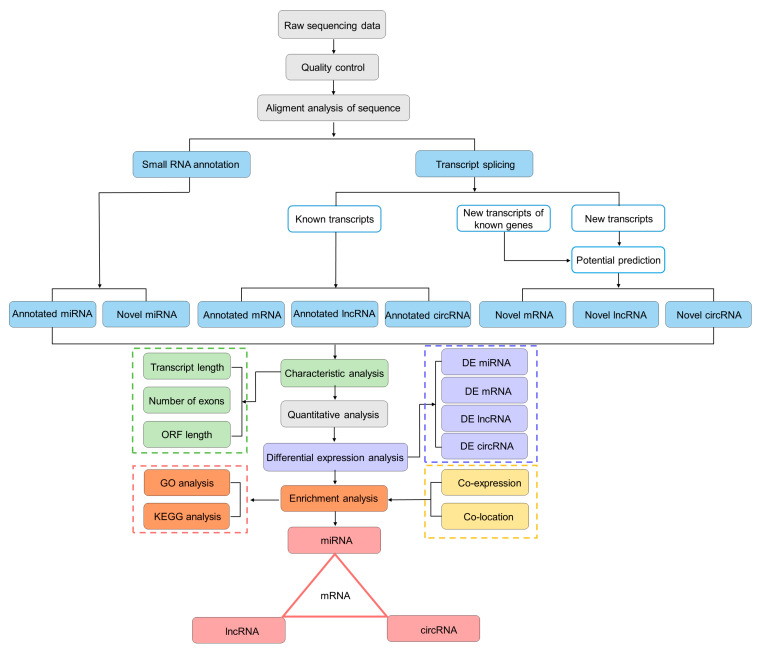
The flowchart of whole-transcriptome association analysis.

In this study, we aimed to obtain a global view of the gill tissue transcriptome (including mRNAs, miRNAs, lncRNAs, and circRNAs) of the *T. rubripes*, to explore which mRNA and ncRNA were involved in *T. rubripes* resisting *C. irritans* infection (Figure 2). Six RNA-seq libraries were constructed. For miRNA sequencing, 93,436,063 reads and 91,896,386 clean reads were obtained from six libraries. For each library with a data volume exceeding 0.76G, the percentage of clean reads in the raw reads was more than 97%. The Q30 values were above 97%. The error rate was 0.01%. After small RNA length screening, 86,636,517 total sRNA readings were produced with more than 0.28 GB. The clean reads for each sample were individually aligned to the specified reference genome, with matching rates between 94.55% and 97.89%. The miRNA underwent length screening, repeat sequence alignment, and exon/intron alignment. The analysis of miRNA lengths revealed a characteristic distribution, predominantly centered at 22 nucleotides (Supplementary Table S2). For sequencing of mRNA, lncRNA, and circRNA, six libraries yielded a cumulative 94.99 GB of clean data, exceeding 15.55 GB per sample, with Q30 base percentages surpassing 90.85%. Each sample’s clean reads were separately mapped to the designated reference genome, yielding alignment rates from 87.21% to 90.52% (Supplementary Table S3). In order to perform feature analysis of lncRNA, transcript length, exon count, and ORF length were analyzed. Based on this, the screened lncRNAs were divided into 29.5% LincRNAs, 28.3% antisense lncRNAs, and 42.2% sense overlapping. In comparing length, ORF length, and exon numbers, both annotated and novel lncRNAs are generally shorter or smaller than mRNA. The ORF length of messenger ribonucleic acid and ribonucleic acid is mostly within 1000, and the lncRNA density reaches 0.0100, while messenger ribonucleic acid is less than 0.0025 (Figure S1).

### 3.3. Expression Profiles and Enrichment Analysis of DEMs

**Figure 3:**
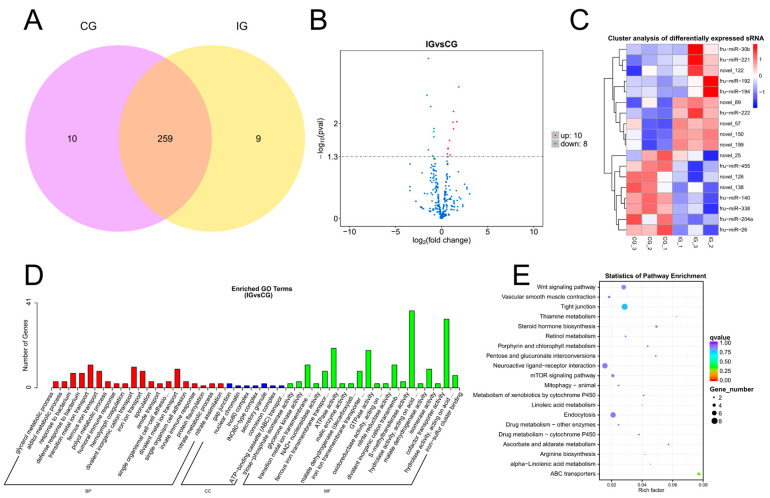
Detection and functional analysis of DEMs associated with *T. rubripes* exposure to *C. irritans* infection. (**A**) Venn diagram of DEMs. (**B**) Volcano map of DEM. No significance is shown in blue, up-regulated DEMs are shown in red, and down-regulated DEMs are shown in green. (**C**) Heat map of DEMs. Identification of DEMs between the CG and IG based on the criteria of padj < 0.05 and |log2FC| > 1. Up-regulated DEMs are depicted in red, while down-regulated DEMs are depicted in blue. (**D**) GO analysis of DEM target genes. (**E**) KEGG pathway analysis of DEM target genes. Statistical significance threshold set at padj < 0.05.

TPM density distribution can comprehensively examine the gene expression patterns of samples (Figure S2A). We conducted a correlation check on gene expression levels between samples (Figure S2B). The Venn shows that 259 DEMs were expressed in both CG and IG, 10 DEMs were specifically expressed in the CG, and 9 DEMs were specifically expressed in the IG (Figure 3A). According to padj < 0.05 and |log2(foldchange)| > 1, 18 DEMs were identified, including 10 known and 8 novel miRNAs. The volcano plot shows the screening of 10 up-regulated DEMs and 8 down-regulated DEMs (Figure 3B). Cluster analysis was performed on differentially expressed genes to obtain a heatmap, which was divided into two clusters (Figure 3C). In the CG, the first type was highly expressed, whereas it exhibited low expression in the IG, with a total of eight differentially expressed miRNAs, such as fru-miR-455, fru-miR-140, fru-miR-338, fru-miR-204a, and fru-miR-26. The second type showed low expression in the control group and high expression in the IG, with a total of ten DEMs, such as fru-miR-30b, fru-miR-221, fru-miR-192, fru-miR-194, and fru-miR-222.

We selected mRNA targets predicted by miRanda and RNAhydrid. A cumulative total of 9623 target genes were obtained. We performed GO and KEGG pathway enrichment analysis of the genes targeted by differentially expressed miRNAs within each experimental group. GO showed that target genes of DEMs were enriched in humoral immune response, innate immune response, and hydrolase activity (Figure 3D). The primary KEGG pathways enriched for the differential target genes in the CG and the IG were the Wnt signaling pathway and the mTOR signaling pathway. These pathways play a crucial role in immune function (Figure 3E).

### 3.4. Expression Profiles and Enrichment Analysis of DELs

**Figure 4:**
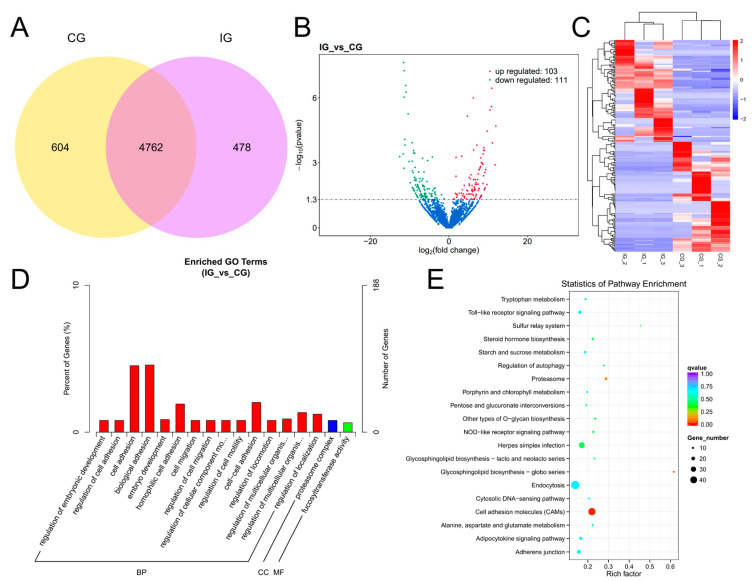
Detection and functional analysis of DELs associated with *T. rubripes* exposure to *C. irritans* infection. (**A**) Venn diagram of DELs. (**B**) Volcano map of DELs. No significance is shown in blue, up-regulated DELs are shown in red, and down-regulated DELs are shown in green. (**C**) Heat map of DELs. Identification of DELs between the CG and IG based on the criteria of padj < 0.05 and |log2FC| > 1. Up-regulated DELs are depicted in red, while down-regulated DELs are depicted in blue. (**D**) GO analysis of DEL target genes. (**E**) KEGG pathway analysis of DEL target genes.

The expression of lncRNAs in each group was stable and the sequencing data were of good quality (Supplementary Figure S3, Supplementary Table S4). Venn analysis revealed that there were a total of 4762 DELs that were common between the IG and CG. Additionally, 604 DELs were expressed in the CG, while 478 DELs were identified in the IG (Figure 4A). A total of 214 significant DELs were identified according to the threshold padj < 0.05. As shown in Figure 4B, the IG showed 103 up-regulated genes and 111 down-regulated genes compared to the CG. Hierarchical clustering analysis as shown in Figure 4C shows that each group had a unique set of lncRNAs that were significantly up-regulated in expression only within each group, and within the other groups were significantly down-regulated in expression. The difference in lncRNA expression within groups was not significant and the trend was consistent. There was a significant difference between groups; the trend of up-regulation and down-regulation was obvious. The results indicated that stimulation of the *C. irritans* infection affected the lncRNA expression pattern in the gills of *T. rubripes*.

Through analyzing the positional relationship and expression correlation between lncRNAs and protein-coding genes, we were able to predict the cis and trans target genes of DELs. This allowed us to gain insight into the potential regulatory roles these lncRNAs may have on the expression of nearby or distant genes. Additionally, we further investigated the functions of these target genes by analyzing their distribution and utilizing GO and KEGG. This comprehensive analysis provided valuable information on the biological processes and signaling pathways that could be impacted by the activity of DELs, shedding light on their potential roles in gene regulation. The GO analysis of co-location is shown in Figure 4D; the target genes of DELs in the IG were annotated to 2801 GO categories compared to the control group, of which 1588 annotations were for biological processes, 395 annotations for cellular components, and 818 to molecular functions. The DELs were predominantly enriched in cell migration, cell adhesion, and proteasome complex. KEGG analysis showed that compared with the CG, the DEL target genes were mainly enriched in TLR signaling pathway, NOD-like receptor (NLR) signaling pathway, p53 signaling pathway, and PPAR signaling pathway (Figure 4E). There were 17 DELs enriched in TLR signaling pathway, 11 DELs enriched in NLR signaling pathway, 36 DELs enriched in p53 signaling pathway, and 38 DELs enriched in PPAR signaling pathway.

### 3.5. Expression Profiles and Enrichment Analysis of DECs

**Figure 5:**
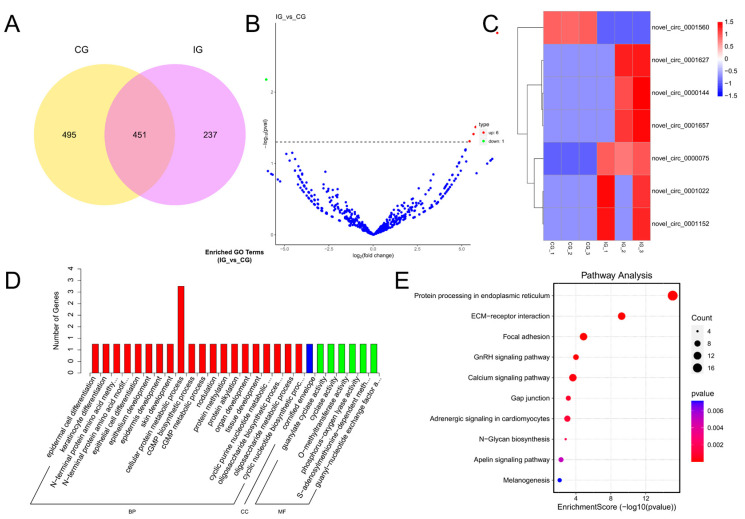
Detection and functional analysis of DECs associated with *T. rubripes* exposure to *C. irritans* infection. (**A**) Venn diagram of DECs. (**B**) Volcano map of DECs. No significance is shown in blue, up-regulated DECs are shown in red, and down-regulated DECs are shown in green. (**C**) Heat map of DECs. Identification of DECs between the CG and IG based on the criteria of padj < 0.05 and |log2FC| > 1. Up-regulated DECs are depicted in red, while down-regulated DECs are depicted in blue. (**D**) GO analysis of DEC target genes. (**E**) KEGG pathway analysis of DEC target genes.

CircRNAs were identified using find_circ with CIRI, and the percentage of circRNA sources was 13% intron, 20% intergenic, and the rest were exon. circRNA expression was present in all groups. An analysis using Venn diagrams revealed that 451 DELs were found between the IG vs. CG, 495 DELs in the CG, and 237 DELs in the IG (Figure 5A). Based on padj < 0.05 and log2(foldchange)| > 1, DECs were screened as in Figure 5B, and the IG showed six up-regulated expressions and one down-regulated expression compared to the CG. Cluster analysis showed that DECs were divided into two groups, novel_circ_0001560 was highly expressed in the CG and expressed to a low degree in the IG. The other group was expressed to a low degree in the CG and highly expressed in the IG (Figure 5C). The target genes of DECs were annotated into 168 GO categories, of which 107 were annotated for biological processes, 20 for cellular components, and 41 for molecular functions (Figure 5D). The DECs were predominantly enriched for cellular protein metabolic process, cGMP biosynthetic process, and phosphorus–oxygen lyase activity. KEGG enrichment analysis connects biological signaling pathways (Figure 5E). Differentially expressed circRNA target genes are linked to metabolic pathways, various cellular processes, and immune response. In the IG compared to the CG, 11 target genes were enriched in the Calcium signaling pathway, three target genes were enriched in the PPAR signaling pathway, and eight target genes were enriched in the Apelin signaling pathway.

### 3.6. Expression Profiles and Enrichment Analysis of DEGs

**Figure 6:**
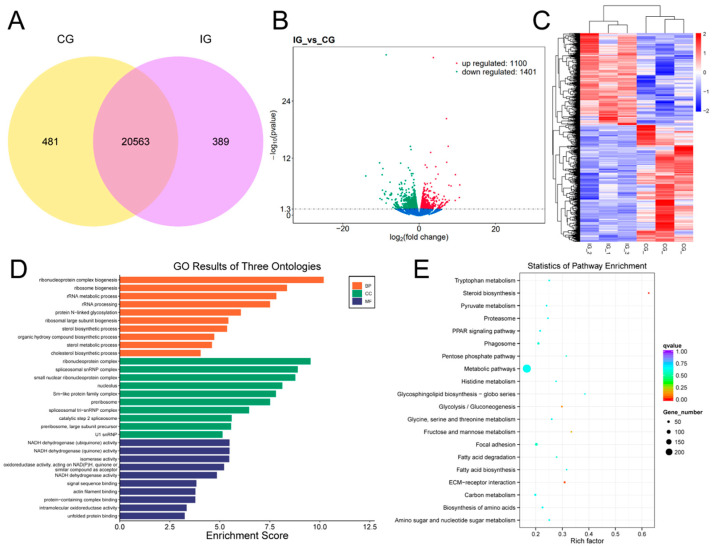
Detection and functional analysis of DEGs associated with *T. rubripes* exposure to *C. irritans* infection. (**A**) Venn diagram of DEGs. (**B**) Volcano map of DEGs. No significance is shown in blue, up-regulated DEGs are shown in red, and down-regulated DEGs are shown in green. (**C**) Heat map of DEGs. Identification of DEGs between the CG and IG based on the criteria of padj < 0.05 and |log2FC| > 1. Up-regulated DEGs are depicted in red, while down-regulated DEGs are depicted in blue. (**D**) GO enrichment analysis of DEG target genes. (**E**) KEGG pathway analysis of DEG target genes.

For the screening of differentially expressed genes, as shown in Figure 6A, 20,563 DELs were identified between the IG and CG. Among these, 481 DELs were found in the CG, while 389 DELs were uniquely expressed in the IG. There were 2501 differentially expressed mRNAs in the IG compared to the CG, with 1100 up-regulated and 1401 down-regulated expressions (Figure 6B). As shown in Figure 6C, the DEGs that were down-regulated in the CG showed a trend of up-regulation in the IG. Furthermore, compared to the CG, the IG DEG is mainly annotated into the GO category for the ribonucleoprotein complex biogenesis, protein N-linked glycosylation, and NADH dehydrogenase activity (Figure 6D). The KEGG pathway analysis is shown in Figure 6E, and the 238 DEGs were enriched in the metabolic pathway, while 19 DEGs were highly expressed in the PPAR signaling pathway.

### 3.7. CeRNA Network Construction

**Figure 7:**
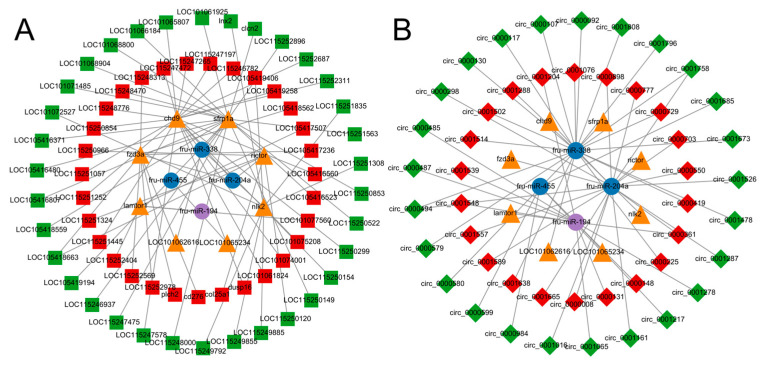
Identification of the competing endogenous RNA network. (**A**) lncRNA-miRNA-mRNA network. (**B**) circRNA-miRNA-mRNA network. The blue circle indicates down-regulated miRNAs, the purple circle indicates up-regulated miRNAs, the yellow triangle indicates mRNAs, the red square indicates up-regulated lncRNAs, the green square indicates down-regulated lncRNAs, the red diamond indicates up-regulated cricRNAs, and the green diamond indicates down-regulated circRNAs.

The differentially expressed miRNAs, lncRNAs, circRNAs and mRNAs were first screened, followed by the prediction of target genes of DEMs enriched in the immune pathway. In addition, DEL and DEC, which have target relationships with target genes and DEMs and are differentially expressed, were also predicted and correlation coefficients were calculated, respectively. Based on the correlation coefficients (Supplementary Materials File S1), using expressed gene pairs with the same miRNA binding sites, a key miRNA-lncRNA-mRNA-ceRNA regulatory network was constructed based on the ceRNA theory by the above DEM, DEL and DEG, revealing that non-coding RNAs play important roles in the immune response of *T. rubripes* to *C. irritans* infection. According to the prediction, six crucial mRNAs were expected to interact with three miRNAs, which would then interact with 64 lncRNAs (Figure 7A). As a result, an immune-related ceRNA network involving lncRNA-miRNA-mRNA interactions was formed, with five core miRNAs, competing with lncRNAs for mRNA interactions. Of these, fru-miR-194, fru-miR-204a, and fru-miR-338 interacted with *lamtor1*, *rictor*, *fzd3a*, *chd9*, *sfrp1a*, and *nlk2* targets. fru-miR-204a-*rictor* up-regulated three lncRNA (LOC115251324, LOC115250966, col25a1) and down-regulated two lncRNA (LOC115250120, LOC105418663). fru-miR-204a-*fzd3a* up-regulated seven lncRNA (cd276, plch2, LOC115248313, LOC105417236, LOC115252978, LOC115252569, LOC105418562), and down-regulated LOC105418663. *rictor* competed with *fzd3a* for LOC105418663. fru-miR-204a-*chd9* interacted with 14 down-regulated lncRNAs and six up-regulated lncRNAs. fru-miR-338-*sfrp1a* up-regulated 2 lncRNAs (LOC115248470, LOC105416523) and down-regulated 19 lncRNAs. Nine lncRNAs participated in the fru-miR-338-*nlk2* interaction, with two up-regulated and seven down-regulated. The up-regulated fru-miR-194 was regulated by LOC115252404, LOC101077560, and LOC105417507 and affected the target gene *lamtor1*. fru-miR-338 was regulated by up-regulated LOC101075208 and LOC105419406, and targeted the *nlk2* gene. In addition, fru-miR-194-*lamtor1* was enriched in the mTOR signaling pathway, while fru-miR-338-*nlk2* was enriched in the Wnt signaling pathway.

Integrating the circRNA-miRNA and the miRNA-mRNA regulatory networks, a total of four key miRNAs can interact with 47 circRNAs, which can subsequently interact with eight mRNAs. Thus, an immune-associated miRNA-circRNA-mRNA-ceRNA network contains eight miRNA-centred interactions with circRNAs competing for mRNAs (Figure 7B). Unlike the lncRNA-miRNA-mRNA network, fru-miR-455-LOC101065234 appeared only in the circRNA-miRNA-mRNA network and four circRNAs were involved in regulation, including three down-regulated circRNAs (circ_0000984, circ_0000599, circ_0001685) and up-regulated circ_0000148. Four up-regulated circRNAs and five down-regulated circRNAs compete with *lamtor* for binding to fru-miR-194. fru-miR-204a is regulated by nine up-regulated circRNAs and five down-regulated circRNAs and targets *rictor*, *fzd3a*, and *chd9*. There are 12 down-regulated circRNAs and eight up-regulated circRNAs down-regulated fru-miR-338 and competed for *sfrp1a*, *nlk2*, and LOC101062616. The fru-miR-204a targeting *chd9* was regulated by circ_0000361, circ_0000494, and circ_0001758 and was enriched in the Wnt signaling pathway. circ_0000361 up-regulated and simultaneously regulated fru-miR-194 expression, and down-regulated circ_0000494 and circ_0001758 regulated fru-miR-338 expression. This suggests that non-coding RNAs play an important role in the immune process of the *T. rubripes* against stimulation of *C. irritans* infection.

### 3.8. lncRNA-circRNA-miRNA-mRNA Construction

**Figure 8:**
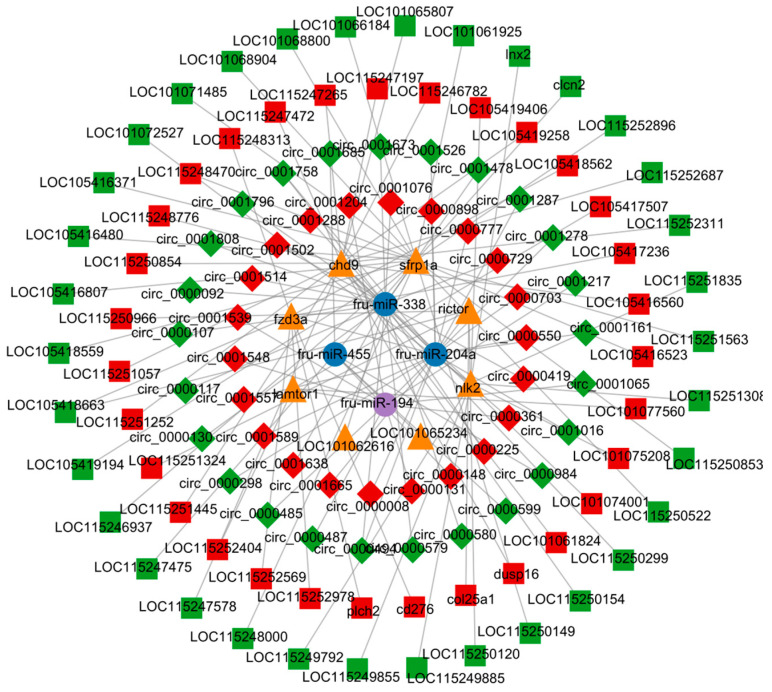
Identification of the lncRNA-circRNA-miRNA-mRNA network.

**Figure 9:**
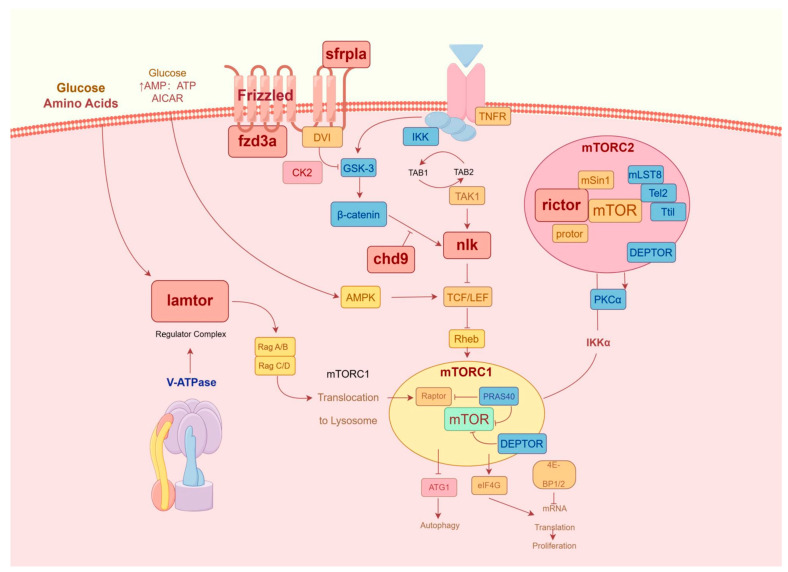
Immune-related pathways of gene interaction.

Combined analysis of lncRNA-miRNA-mRNA and circRNA-miRNA-mRNA revealed an interaction network consisting of 64 lncRNAs, 47 circRNAs, 4 miRNAs, and 8 mRNAs (Figure 8). At the same time, in order to further clarify the anti-*C. irritans* immune mechanism of *T. rubripes*, the pathway relationship enriched by the screened immune genes is shown in Figure 9. These were mainly enriched in the Wnt signaling pathway and mTOR signaling pathways. Among them, *chd9*, *nlk2*, *sfrp1a*, and *rictor* gene expression was down-regulated and *fzd3a* and *lamtor1* gene expression was up-regulated. Other genes were screened and linked to immune genes. fru-miR-194-*lamtor1* interacted with three up-regulated lncRNAs such as LOC115252404, LOC101077560, LOC105417507, and nine circRNAs such as circ_0000487, circ_0000485, circ_0001539, which constitute a ceRNA regulatory network, in which five circRNAs were down-regulated and four circRNAs were up-regulated. fru-miR-204a, with 16 circRNAs and 32 lncRNAs, regulated the expression of *rictor*, *fzd3a*, and *chd9*. Among them, there were 24 up-regulated lncRNAs and 9 up-regulated circRNAs, 8 down-regulated lncRNAs and 7 down-regulated circRNAs, and fru-miR-204a expression was down-regulated. In addition, fru-miR-204a competed with fru-miR-194 for binding to circ_0000361, and competed with fru-miR-338 for binding to circ_0000494 and circ_0001758. fru-miR-338 targeted 3 DEGs (*sfrp1a*, *nlk2*, and LOC101062616), and regulated the expression of 20 circRNAs and 30 lncRNAs. Among them, there were 4 up-regulated lncRNAs and 8 up-regulated circRNAs, 26 down-regulated lncRNAs and 12 down-regulated circRNAs, and fru-miR-338 expression was down-regulated. However, mRNA LOC101062616 did not match significantly differentially expressed lncRNAs. fru-miR-204a targeted *fzd3a* and *chd9* was enriched in the Wnt signaling pathway, while fru-miR-204a targeted *fzd3a* and *rictor* was enriched in the mTOR signaling pathway. fru-miR-204a and *fzd3a* were simultaneously involved in two important immune pathways, so we hypothesized that the interactions between fru-miR-204a and *fzd3a* play an important role in the immune response against *C. irritans* in *T. rubripes*. Since LOC105418663 was involved in both *rictor* and *fzd3a* expression, and circ_0000361 regulated both fru-miR-204a and fru-miR-194, a lncRNA-circRNA-miRNA-mRNA ceRNA network composed of LOC105418663-circ_0000361-fru-miR-204a-*fzd3a* was formed. The only up-regulated key fru-miR-194 targeted *lamtor1* was enriched in the mTOR signaling pathway.

### 3.9. Validation of ncRNAs

**Figure 10:**
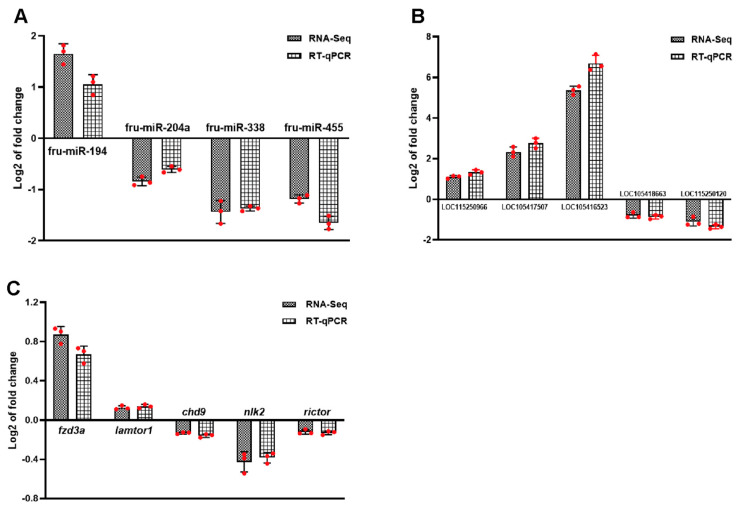
Identification of ncRNAs by qRT-PCR. (**A**) DEMs. (**B**) DELs. (**C**) DEGs. Log2 FC was expressed as mean ± SD. n = 3. The statistical significance of all genes reached padj < 0.05.

To further validate the accuracy of the RNA-seq data, four DEMs, five DELs, and five DEGs in the ceRNA were randomly identified. Correlation analysis was performed (Supplementary Figure S4). The findings showed that the RNA molecules in qRT-PCR followed the same trend as those in RNA-seq, and the results were stable (Figure 10A–C).

## 4. Discussion

Currently, parasites are the main problem affecting fish farming. Parasites can even use immune evasion to invade the host, such as avoiding immune system surveillance, molecular mimicry, immunosuppression, immune regulation, and manipulation of the host’s endocrine and nervous systems [26,27]. The host can have different physiological responses to parasites at different stages of development. The immune response of *Salmo trutta* to developing parasites is that most immune-related genes are up-regulated, while in *Oncorhynchus mykiss* at the later stage of parasite development, the immune response is weakened and metabolic processes are increased [28,29]. Related studies on fish gill tissues before and after infection with *Ichthyophthirius multifiliis* found that some of the up-regulated differentially expressed genes were enriched in pathways related to inducing immune responses [30]. However, there are few relevant studies on *C. irritans*.

MicroRNA (miRNA) is a type of short, single-stranded, ncRNA molecule that can regulate gene expression by binding to the 3′UTR of target genes, leading to protein translation obstruction or mRNA degradation [31]. Previous studies have shown that a large number of fish miRNAs are involved in immune responses associated with pathogen infections, for example, infectious pancreatic necrosis virus (IPNV)-induced Atlantic salmon (*Salmo salar*) [32], tilapia lake virus (TiLV)-induced tilapia (*Oreochromis* spp.) [33], and Japanese flounder (*Paralichthys olivaceus*) induced by *Vibrio anguillarum* [34]. These miRNAs are mainly enriched in the TLR signaling pathway, the NLR signaling pathway, the Wnt signaling pathway, and the mTOR signaling pathway. They are involved in regulating innate and acquired immune responses by regulating immune cell activity, inflammatory response, antimicrobial protein expression, and immune-related signaling pathways. Additionally, fish primarily rely on their innate immune system to resist pathogen invasion, with the gill mucosa being a critical component. As the first line of defense, gills are often the initial site of pathogen attack during infection. Therefore, elucidating the immune response mechanism against infection is crucial to investigate the essential signaling pathways and immune genes in the gill mucosa.

MiR-194 is a newly identified molecule that plays a significant role in various biological processes. It is extensively involved in the regulation of innate immunity, which is the first line of defense against pathogens. Additionally, miR-194 is critical in controlling apoptosis, the process of programmed cell death, essential for maintaining cellular homeostasis and eliminating damaged or unneeded cells. Furthermore, miR-194 has a substantial impact on inflammation, a complex biological response to harmful stimuli such as infections or injuries. Lastly, miR-194 is deeply integrated into the mechanisms of autophagy. Previous research has indicated that miR-194 has dual characteristics in a variety of diseases. In the inflammatory response, miR-194 has been identified as a promising anti-inflammatory agent [35]. In this study, we found that miR-194 was significantly up-regulated in the case of *C. irritans* infection, which was consistent with results from studies on inflammation in *Danio rerio*, *Cyprinus carpio*, and *Mus musculus* [36]. Two weeks post-recovery from gossypol poisoning, the expression of miR-194 in *C. carpio* was significantly reduced. This suggests that miR-194, as a new inflammatory response regulator, actively participates in the immune response. Meanwhile, miR-194 was significantly up-regulated in zebrafish infected with *V. harveyi*, and overexpression of miR-194 affected LPS-induced increase in immune-related genes [37]. It can suppress the activation of genes associated with inflammation caused by lipopolysaccharide, such as IL-1 and TNF-α [38,39,40]. MiR-194 plays a role in the NF-κB pathway by interacting with TRAF6 [39], TRIM23, and C21ORF91, and several other target genes [41]. In addition, there is evidence that circ_0001278 and circ_0001204 are involved in the regulation of cancer progression, and circ_0001204 can be used as a new biomarker for tuberculosis diagnosis [42,43,44]. The results of this study suggest that circ_0001278 and circ_0001204 may interact with miR-194 and regulate the downstream target gene *lamtor1*. The target gene *lamtor1* is closely related to cell growth, autophagy and immune regulation [45,46]. Wu et al. found that *lamtor1* could reduce MHC-II expression, thereby decreasing the anti-tumor response of T cells [47], suggesting that *lamtor1* may have an oncogenic role. Knockdown of *lamtor1* expression was reported to reduce the activity of non-small cell lung cancer (NSCLC) cells. This is consistent with our results, which showed that fru-miR-194 was up-regulated and targeted *lamtor1* was down-regulated to resist stimulation of *C. irritans* infection. On the other hand, *lamtor1* functions as a modulator of the mTORC1 protein. A decrease in *lamtor1* expression results in the suppression of mTORC1 activity within retinal pigment epithelial cells [48]. This change has also been observed in NSCLC cells, where the attenuation of *lamtor1* led to reduced levels of phosphorylation of mTOR expression. *Lamtor1* facilitates the activation of mTORC1, influencing the advancement of colorectal cancer (CRC) induced by inflammation [49]. Moreover, the activation of mTORC1 relies heavily on the ubiquitination of *lamtor1*, making it a viable target for therapeutic interventions in various diseases.

miR-455 is an emerging molecule that regulates the immune response. miR-455-3p mainly targets genes related to apoptosis and inflammation. Reversing inflammation development and apoptosis induced by circ_102049 knockdown may be achieved through the down-regulation of miR-455-3p [50]. Interestingly, miR-455 expression in gill tissues was reduced after infection with *C. irritans*. We speculate that miR-455 is similarly involved in modulating anti-inflammatory responses in *T. rubripes*. miR-455 was down-regulated during *Trionyx sinensis* hemorrhagic syndrome virus infection, which was also consistent with our results. Research has demonstrated that miR-455 is crucial for *Nile tilapia’s* response to cold stress and also influences the rhythmic expression of skeletal muscle in *Carassius auratus*. However, another study showed that overexpression of miR-455-3p led to attenuation of excessive stress-induced cardiac hypertrophy. The results indicate that miR-455 may have a dual function in the inflammatory pathway. In this study, miR-455 targeted a key gene that was associated with four circRNAs, with circ_0000148 being up-regulated and the others down-regulated. Of these, circ_0000984 can up-regulate the expression of CDK6 gene by competitively binding to miR-106b, thereby playing a role in the growth and metastasis of tumor cells [51]. CircRNA is non-coding closed-loop RNA produced by pre-mRNA reverse splicing [52]. Existing literature has shown that circRNA can regulate inflammatory responses by affecting the level of downstream targets [53]. Additionally, because of its covalent closed-loop structure, circRNA is more stable than linear RNA and can be directly released from cells through extracellular vesicles. Therefore, circRNA, as a candidate for therapeutic vaccines, has potentially important functions in gene expression regulation. Based on the results of transcriptome analyses, we believe that the mechanism of miR-455 in the inflammatory response in fish needs to be further investigated.

In related studies of teleost, miR-338 has been shown to participate in regulating the immune response. Studies have shown that miR-338 may have protective or harmful effects in different diseases. For example, miR-338 can inhibit angiogenesis by targeting vascular endothelial growth factor (VEGF) [54]. miR-338 inhibits the transition of gastric cancer cells by inactivating the Wnt signaling pathway [55]. Another study showed that miR-338 has an inhibitory effect on RCC [56,57]. Moreover, the overexpression of miR-338 reduced LPS-induced damage to 16HBE cells [58]. miR-338 targeted at least three key immune-related genes after *Oncorhynchus mykiss* was infected with hematopoietic necrosis virus (IHNV). In this study, fru-miR-338 also targeted three key genes, including *nlk2*, *sfrp1a*, and LOC101062616. Moreover, the expression of fru-miR-338 decreased after infection with *C. irritans*, suggesting that it may participate in the immune response against *C. irritans* and alleviate fish infection. Additionally, studies have shown that miRNA can affect the stability of lncRNA by interacting with lncRNA [59,60]. Notably, we predicted and verified the binding relationship between fru-miR-338 and lncRNA, which further suggested that fru-miR-338 and lncRNA interacted in the anti *C. irritans* response. CircRNAs can regulate inflammatory response by affecting the level of downstream targets [53]. At present, circRNAs in aquatic organisms against *C. irritans* or other parasitic diseases, such as circMIB2, novel_circ_002325, have been discovered and identified. Our results showed that circRNA was abnormally elevated in the infection group of *T. rubripes*, and fru-miR-338 targeted nlk2 through circ_0001502 to ultimately inhibit cell apoptosis, thereby affecting the Wnt signaling pathway. miR-338 can reduce the inflammatory damage to cells, and circ0038467 plays a role through the molecular sponge of miR-338 [61]. Circ_0026579 has a similar effect in targeting miR-338 [62]. In addition, among the 20 circRNAs that have a targeted relationship with fru-miR-338, circ_0000419 and circ_0000729 have been shown to play a certain promoting role in the proliferation, migration, and other developmental processes of cancer cells [63,64]. This is consistent with our research findings, as both circ_0000419 and circ_0000729 in the infected group showed up-regulation in the interaction between circ_0000419-fru-miR-338 and circ_0000729. This indicated that the expression of circ_0000419 and circ_0000729 was positively correlated with fish before and after infection. Additionally, studies have shown that hsa_circ_0000092 has a targeted relationship with microRNA-338-3p. The interaction between hsa_circ_0000092 and microRNA-338-3p can regulate HN1 to affect life processes such as cell proliferation, invasion and angiogenesis [65]. Similarly, our research findings suggest that circ_0000092 interacts with fru-miR-338 and forms axial relationships with three target genes. Among them, *nlk2* and *sfrp1a* have been shown to be enriched in the Wnt signaling pathway and involved in innate immune response. In summary, the pathogenesis of *C. irritans* is complex and multifactorial, including pathophysiological factors such as immune dysfunction and inflammatory response, which ultimately lead to organ dysfunction [66]. Although the relationship between circRNA, fru-miR-338, and mRNA is not entirely clear, our results still provide a new theoretical basis for anti-*C. irritans* infection.

In this research, the low expression of fru-miR-338 targeted *nlk2*, *sfrp1a* and LOC101062616 mediated immune response, indicating that fru-miR-338 regulation can drive immune response progression. The key genes *nlk2* and *sfrp1a* have been confirmed to be enriched in the Wnt signaling pathway, contributing to the innate immune response. NLK, a protein kinase known as Nemo-like kinase, plays a crucial role in both the Wnt signaling pathway and immune response [67,68,69,70,71,72]. It is reported that *nlk* is a regulator of primary neurogenesis in the neural plate, widely expressed in early development, and acts as a positive regulator of Wnt signaling [73]. *nlk* not only participates in the development of the nervous system in vertebrates but is also involved in various diseases [4,5,6,7,8]. *nlk2* plays a crucial role in viral infections in mammals. Specifically, when HEK293T cells are infected with SEV, *nlk2* suppresses the production of ISG56, IFN-β, and ISG15 [74,75]. *nlk* positively regulates Wnt signal transduction in mammalian neural progenitor cell (NPC)-like cell lines, negatively regulates HEK293 and HeLa cells, and inhibits β-Catenin-mediated transcription [67,76]. Additionally, *nlk* gene sequences have been reported in many aquatic organisms. For example, the *nlk2* of grass carp (*Ctenopharyngodon idella*) responds to grass carp reovirus (GCRV) stimulation and is highly expressed in the gill and kidney, suggesting that *nlk2* is involved in the immune response [77]. Generally, few studies focus on the immune function of *nlk2* in teleost. However, it is worth noting that *nlk2* is highly conserved from worms to humans [78], suggesting that *nlk2* is the target gene of fru-miR-338 in this study. fru-miR-338 is down-regulated to regulate the expression of *nlk2* and innate immune response, revealing a new role for *nlk2* in activating the Wnt signaling pathway. In addition to *nlk*, the key gene *sfrp* can bind Wnt ligands, regulate Wnt signaling and signal through Wnt receptors. *sfrp1* has been identified in medaka, zebrafish, and xenopus [79,80] as a Wnt antagonist related to frizzled, which is expressed in the central nervous system and intestine of zebrafish [81]. Functional analysis of *Drosophilid* showed that *sfrp* could act as both an inhibitor and promoter, affecting signal activation [82]. However, there are no reports about the expression of *sfrp1* in *T. rubripes*.

miR-204a plays a crucial role in regulating cytokine levels within cellular structures. MiRNA generally regulates its targets through translation inhibition and mRNA instability [83,84]. In the immune system, miRNA is frequently associated with abnormal expression [85]. Currently, miR-204 has been confirmed to be dysregulated in several cancer cells. For example, miR-204 overexpression can trigger cell apoptosis [86]. The overexpression of miR-204 in macrophages can inhibit TLR4/JNK signal transduction, reduce pro-inflammatory cytokine release, and consequently inhibit macrophage inflammation [87]. Furthermore, studies have confirmed miR-204’s role in regulating the proliferation and metastasis of cancer cells [88,89]. Marco Galasso et al. found that, in vitro, miR-204 has a significant effect on melanin [90]. miR-204 is involved in several inflammatory suppression processes and has become a potential therapeutic target for some diseases. CircRNAs with targeting relationships with fru-miR-204a play an important role in regulating key genes. Studies have shown that circ_0001589 and circ_0001665 are significantly up-regulated in clinical tissue samples and cell lines, and they regulate tumor cell proliferation, invasion, and apoptosis [91,92]. The ceRNA network showed that circ_0001589 and circ_0001665 interacted with fru-miR-204a and regulated three target genes, respectively, and were highly expressed in infected tissues. In addition, the expression of some circRNAs was down-regulated, such as circ_0001287, circ_0001016, circ_0001526, and circ_0001161. Circ_0001287 has been shown to be down-regulated in NSCLC tissues, and its overexpression inhibits proliferation, migration, and invasion, and can be involved in the regulation of NSCLC cell proliferation and migration by indirectly modulating PTEN expression [93]. Similarly, circ_0001016 has been shown to promote the growth of prostate cancer cells by targeting miR-23a during cancer progression [94]. This evidence indicates that non-coding RNAs are important in the development of the disease. It has also been reported that the ceRNA network of *Sebastes schlegelii* after infection with *Aeromonas salmonicida* shows up-regulation of circ_0000298 expression and a positive correlation with infection time [95]. This is consistent with our research findings. Therefore, circ_0000298 may play an important role in disease immunity in fish. There are limited studies on the interaction between miR-204 and circRNAs together in the immune response of fish. Our research revealed a significant decrease in the expression of miR-204a in the gills of *T. rubripes* following infection with *C. irritans*, aligning with findings from prior investigations [96]. In addition, transcriptome data analysis showed that miR-204 simultaneously targeted three key genes, *rictor*, *fzd3a*, and *chd9*. *rictor* was enriched in the mTOR signaling pathway, *chd9* was enriched in the Wnt signaling pathway, and *fzd3a* was enriched in both the mTOR and Wnt signaling pathways.

The conventional understanding is that Wnt signaling is primarily mediated through seven transmembrane proteins belonging to the frizzled (Fzd) family and the low-density lipoprotein receptor-related protein as co-receptors, which subsequently trigger the activation of the canonical Wnt signaling pathway [97,98]. Deletion of *fzd3a* may lead to disorder in the Wnt signaling pathway [99]. It has been reported that the xenopus’s *fzd3* may inhibit typical Wnt signaling by recruiting transfer-related kinases [100]. This shows that the *fzd3* signal has significant biological importance for the common neural circuit across species. On the other hand, knocking down *fzd3* could inhibit the growth and metastasis of melanoma cells [101]. The ncRNAs (such as lncRNA and miRNA) can directly or indirectly target *fzd3*, regulate the Wnt signaling pathway, and affect the proliferation and metastasis of cancer cells [102,103,104]. However, the relationship between *fzd3*-Wnt signaling and the fish immune response has not been reported. In this study, eight lncRNAs associated with *fzd3* were identified, which together with miR-204a constituted the lncRNA-miRNA-mRNA network. This is significant for further screening of functional lncRNAs. Chromodomain helicase DNA binding 9 (*chd9*) is a regulatory factor that facilitates chromatin structural reorganization [105]. The function of *chd9* in human cancer is controversial, and it is considered to be an oncogene in renal cell carcinoma [106] or a tumor suppressor gene in colorectal cancer (CRC) [107]. The role of *chd9* in the immune response to *C. irritans* infection in *T. rubripes* remains uncharacterized. Through bioinformatics analysis, *chd9* has been found to have a binding site for fru-miR-204a. After infection with *C. irritans*, the expression of fru-miR-204a in the gill of *T. rubripes* was down-regulated, while the expression of *chd9* was up-regulated. This is consistent with the role of *chd9* in CRC. Meanwhile, circRNA can act as a sponge to absorb miRNA [87]. Reports indicate that circpdzd8 up-regulates *chd9* and promotes cancer progression by sponging miR-197-5p [108]. In this study, fru-miR-204a regulates the Wnt signaling pathway by binding to *chd9* and simultaneously targeting multiple circRNAs. In conclusion, the combination of *chd9* and fru-miR-204a can serve as a potential therapeutic target, and inhibiting *chd9* expression may be a key mechanism in combating *C. irritans* infection.

The mTOR pathway is essential in controlling the differentiation processes of both innate and adaptive immune cells [109,110]. The role of mTOR in immune cell activation and functional characteristics has been well-established [111]. Similarly, we observed that miR-204a was down-regulated and targeted *rictor* after infection with *C. irritans*. As the core component of mTORC2, *rictor* plays a role in amino acid sensing [112]. *rictor* affects intestinal inflammation in hybrid grouper [113]. Knockdown of *rictor* can induce apoptosis in breast cancer cells, inhibit cell migration and metastasis [114,115], and alleviate pancreatic tumor occurrence [115]. miR-16 and let-7 target *rictor* and regulate the induction of T cells [116]. Therefore, compared to the CG group, regulating the key gene *rictor* in the mTOR pathway by down-regulating miRNA expression is an important immune process in *T. rubripes* against infection. The mTOR pathway integrates multiple environmental signals [117]. Dysregulation of the mTOR pathway has been found in many diseases [118,119,120]. Therefore, targeting the mTOR represents a potential therapeutic strategy [121]. Concurrently, the immune mechanism in *T. rubripes* was induced by *C. irritans*. A total of 16 circRNAs and five lncRNAs combined to form two ceRNA networks, which regulate the mTOR pathway through miR-204a, thereby resisting infection. The research indicates that miR-204a is a crucial component in the immune response mechanism of marine fish to *C. irritans*. In this study, the CG and the IG were mainly enriched in the Wnt signaling pathway and mTOR signaling pathway, which have immune functions. We speculate that immune pathway signals are activated when resisting *C. irritans* infection. This discovery offers a key theoretical foundation for future advancements in vaccine development in related areas.

## 5. Conclusions

In this study, the expression profile of ncRNA after infection with *C. irritans* by *T. rubripes* was comprehensively analyzed. We identified 18 DEMs, 214 DELs, 7 DECs, and 2501 DEGs, and performed functional enrichment analysis. We comprehensively screened and analyzed ncRNAs related to immune pathways including the Wnt signaling pathway and mTOR signaling pathway, and constructed lncRNA-miRNA-mRNA and circRNA-miRNA-mRNA networks. Based on this, we constructed an integrated network of lncRNA-circRNA-miRNA-mRNA and predicted the LOC105418663-circ_0000361-fru-miR-204a-fzd3a axis, further revealing ceRNA function and providing new insights into and support for the study of immune mechanisms related to ncRNAs in *T. rubripes*.

## Data Availability

The original contributions presented in the study are included in the article and Supplementary Materials; further inquiries can be directed to the author.

## Supplementary Materials

The following supporting information can be downloaded at: https://www.mdpi.com/article/10.3390/biology13100788/s1, File S1: Correlation Coefficients; File S2: Supplementary methods and materials; Table S1: Primers used in real-time PCR for validation, Table S2: Summary of microRNA sequencing data, Table S3: Summary of lncRNA sequencing data; Table S4: Reference genome mapped information of LncRNA; Table S5: Reference genome mapped information of miRNA; Figure S1: Statistics on the length distribution of total sRNA fragments and lncRNA Characteristics, Figure S2: Analysis of miRNA expression levels. Figure S3: FPKM expression distribution, Supplementary Figure S4: Correlation analysis between RNA-seq and qRT-PCR.
