# Supplementary material for: Whole-Transcriptome Analysis Reveals Potential CeRNA Regulatory Mechanism in Takifugu rubripes against Cryptocaryon irritans Infection
Source: Biology (Basel). 2024 Oct 1;13(10):788. doi: 10.3390/biology13100788 (PMC11504436; doi:10.3390/biology13100788)
Supplement: Supplementary file 1 [file biology-13-00788-s001.zip › Supplementary figure and table.pdf]

Supplementary Table S1 Primers used in real-time PCR for validation

Gene	Primer Sequence	Purpose
fzd3a	F GGACGACCGCCTGCCTTAC	qRT-PCR
	R TGCTGCTGTGCCTGGACTG	
lamtor1	F TATTCAGATATTCAGCAGGTGTCCAAG	qRT-PCR
	R CCAGTTCTTCCTTCGCATCCAC	
chd9	F TTCATCATCGTCTTCCTCTTCATCATC	qRT-PCR
	R GTGTCTCATCTTGTGTAGTGCTCAG	
nlk2	F TTCCAAGCCCAGAGTCCCATCC	qRT-PCR
	R CTTCGCACGCCGTCCTCATG	
rictor	F AGGCTACGCTACGCTGAAGAG	qRT-PCR
	R CATGGTGATGGTGTCCGTGAAC	
β-actin	F ATCCGTAAGGACCTGTATGC	qRT-PCR
	R AGTATTTACGCTCAGGTGGG	
LOC115250966	F CTTTCGGTTTGCTTTCTTATTGCTTTG	qRT-PCR
	R TTGTTCGGCGGCACCATTTC	
LOC105417507	F AACCGCTGTTCATTCATCTCTTAGTAG	qRT-PCR
	R CATCCACTCTCCGTCTCCCATC	
LOC105416523	F AGGAGGATTATGGTACAGCGATGG	qRT-PCR
	R GCACAGCCACACTCACACTTG	
LOC105418663	F CTCCAGTTGACGCTACAGATGTG	qRT-PCR
	R GTAATGGTGGCAATGTCTCCTGATAG	
LOC115250120	F CCTGCTCCTGGTGGCTACTG	qRT-PCR
	R GATTCCCTCATCATCGGCAACTG	
fru-miR-194	CGTGTAACAGCAACTCCATGTGG	qRT-PCR
fru-miR-204a	GCTTCCCTTTGTCATCCTATGCCT	qRT-PCR
fru-miR-338	CCGTCCAGCATCAGTGATTTTGTTG	qRT-PCR
fru-miR-455	GTCTATGTGCCCTTGGACTACATCG	qRT-PCR
U6	F CTCGCTTCGGCAGCACA	qRT-PCR
	R AACGCTTCACGAATTTGCGT	

Supplementary Table S2 Summary of microRNA sequencing data

Sample name	Raw Reads	Raw Bases(G)	Clean Reads	Mapping rate(%)	Q30	Error rate
CG_1	15663363	0.783G	15355876	98.04%	97.20%	0.01%
CG_2	15682361	0.784G	15233199	97.14%	97.28%	0.01%
CG_3	15370415	0.769G	15179095	98.76%	97.48%	0.01%
IG_1	15570909	0.779G	15379158	98.77%	97.40%	0.01%
IG_2	15606079	0.780G	15459555	99.06%	97.29%	0.01%
IG_3	15542936	0.777G	15289503	98.37%	97.20%	0.01%

Supplementary Table S3 Summary of lncRNA sequencing data

Sample name	Raw Reads	Raw Bases(G)	Clean Reads	Mapping rate(%)	Q30	Error rate
CG_1	105307792	15.80	104752894	88.69%	94.38	0.01%
CG_2	107804248	16.17	107165878	89.42%	94.46	0.01%
CG_3	107124576	16.07	106728746	87.21%	94.35	0.01%
IG_1	104245824	15.64	103683634	87.37%	90.85	0.01%
IG_2	105903864	15.89	105323158	90.52%	93.94	0.01%
IG_3	105965904	15.89	105637388	88.19%	93.51	0.01%

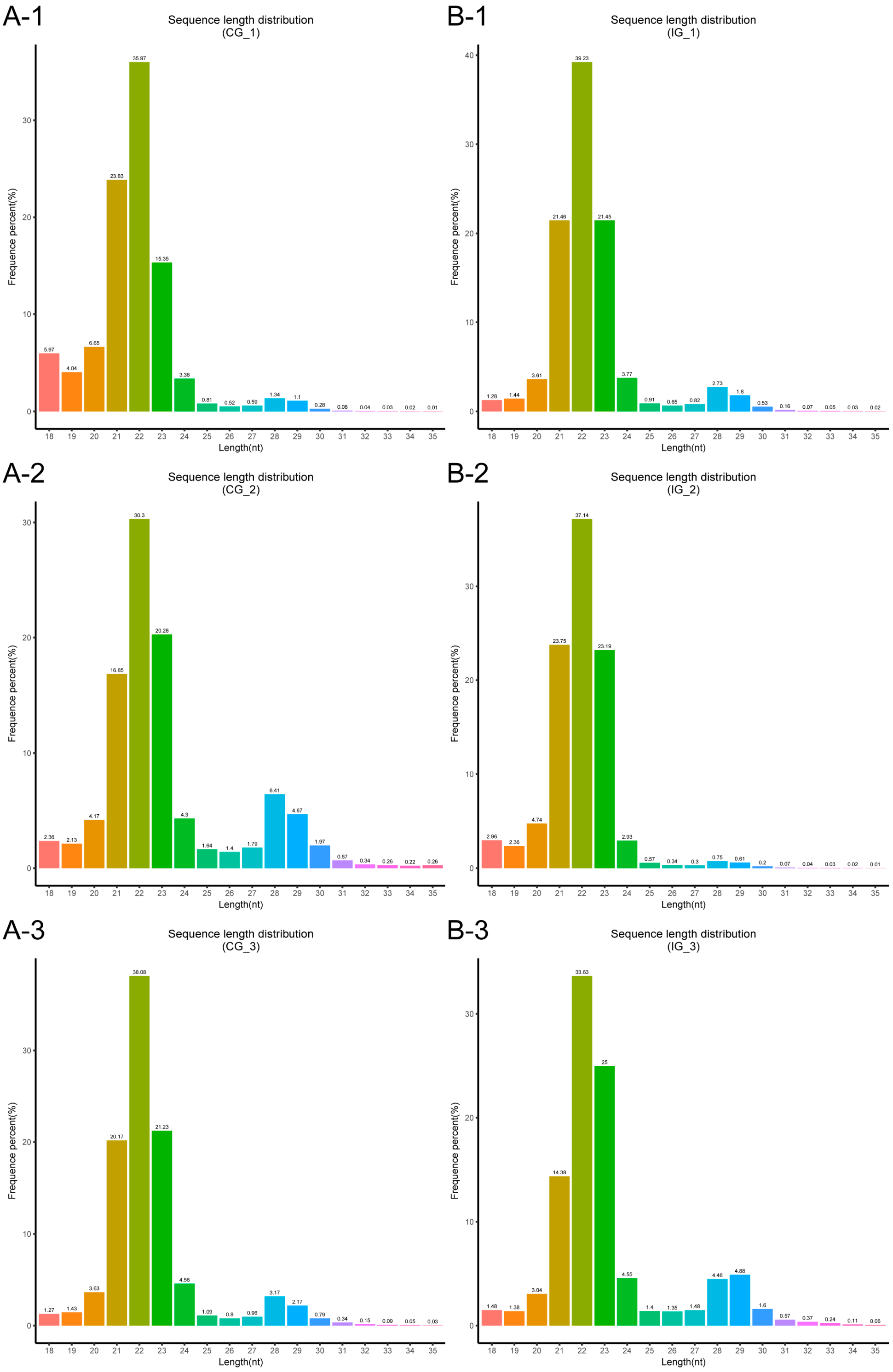
A-1
Sequence length distribution
(CG_1)
Frequence percent(%)
Length(nt)
0
10
20
30
18 19 20 21 22 23 24 25 26 27 28 29 30 31 32 33 34 35
5.97 4.04 6.65 23.83 35.97 15.35 3.38 0.81 0.52 0.59 1.34 1.1 0.28 0.08 0.04 0.03 0.02 0.01
B-1
Sequence length distribution
(IG_1)
Frequence percent(%)
Length(nt)
0
10
20
30
40
18 19 20 21 22 23 24 25 26 27 28 29 30 31 32 33 34 35
1.28 1.44 3.61 21.46 39.23 21.45 3.77 0.91 0.65 0.82 2.73 1.8 0.53 0.16 0.07 0.05 0.03 0.02
A-2
Sequence length distribution
(CG_2)
Frequence percent(%)
Length(nt)
0
10
20
30
18 19 20 21 22 23 24 25 26 27 28 29 30 31 32 33 34 35
2.36 2.13 4.17 16.85 30.3 20.28 4.3 1.64 1.4 1.79 6.41 4.67 1.97 0.67 0.34 0.26 0.22 0.26
B-2
Sequence length distribution
(IG_2)
Frequence percent(%)
Length(nt)
0
10
20
30
18 19 20 21 22 23 24 25 26 27 28 29 30 31 32 33 34 35
2.96 2.36 4.74 23.75 37.14 23.19 2.93 0.57 0.34 0.3 0.75 0.61 0.2 0.07 0.04 0.03 0.02 0.01
A-3
Sequence length distribution
(CG_3)
Frequence percent(%)
Length(nt)
0
10
20
30
18 19 20 21 22 23 24 25 26 27 28 29 30 31 32 33 34 35
1.27 1.43 3.63 20.17 38.08 21.23 4.56 1.09 0.8 0.96 3.17 2.17 0.79 0.34 0.15 0.09 0.05 0.03
B-3
Sequence length distribution
(IG_3)
Frequence percent(%)
Length(nt)
0
10
20
30
18 19 20 21 22 23 24 25 26 27 28 29 30 31 32 33 34 35
1.48 1.38 3.04 14.38 33.63 25 4.55 1.4 1.35 1.48 4.46 4.88 1.6 0.57 0.37 0.24 0.11 0.06

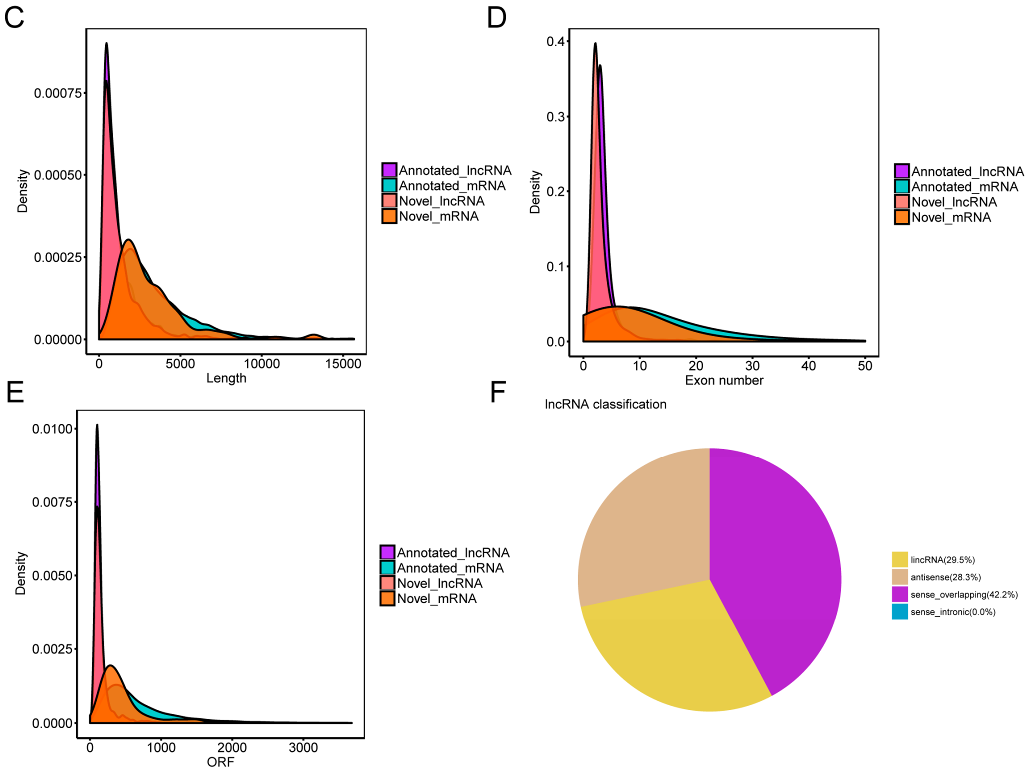

Supplementary Figure S1. Statistics on the length distribution of total sRNA fragments and lncRNA Characteristics. (A) length distribution of CG. (B) length distribution of IG. The horizontal axis represents the length of reads, and the vertical axis represents the proportion of reads of that length. (C) Transcript length. (D) number of exons. (E) ORF length. (F) LncRNA type.

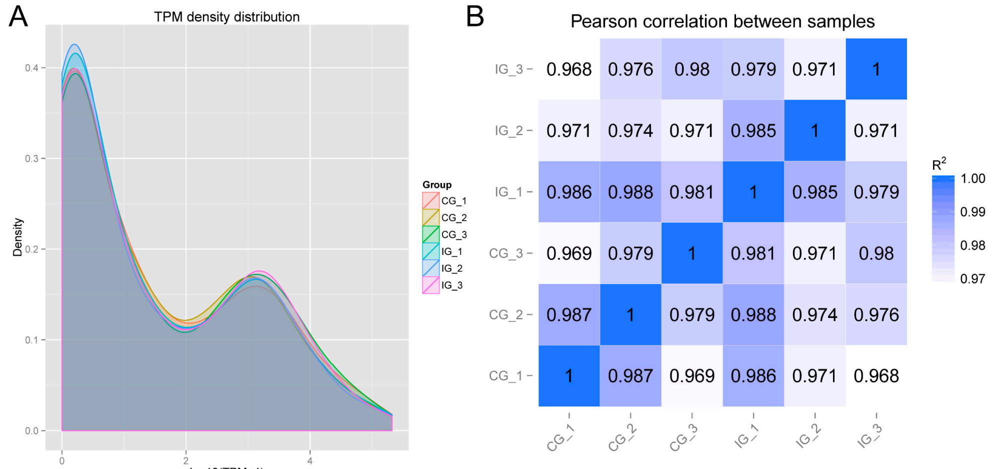

Supplementary Figure S2. Analysis of miRNA expression levels (A) TPM density. (B) Expression level correlation.

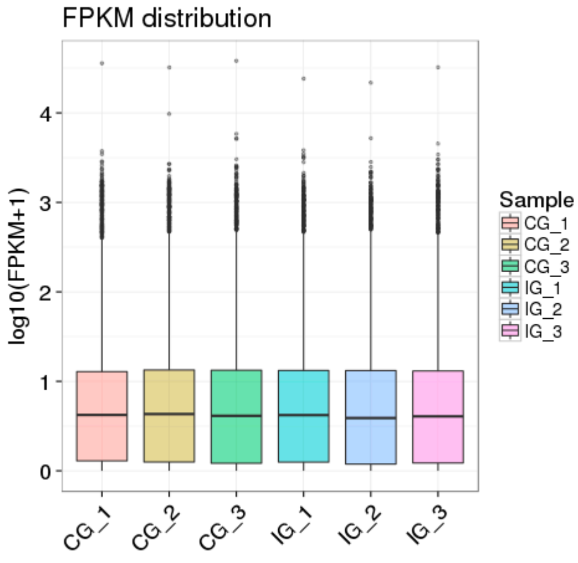

Supplementary Figure S3. FPKM expression distribution.

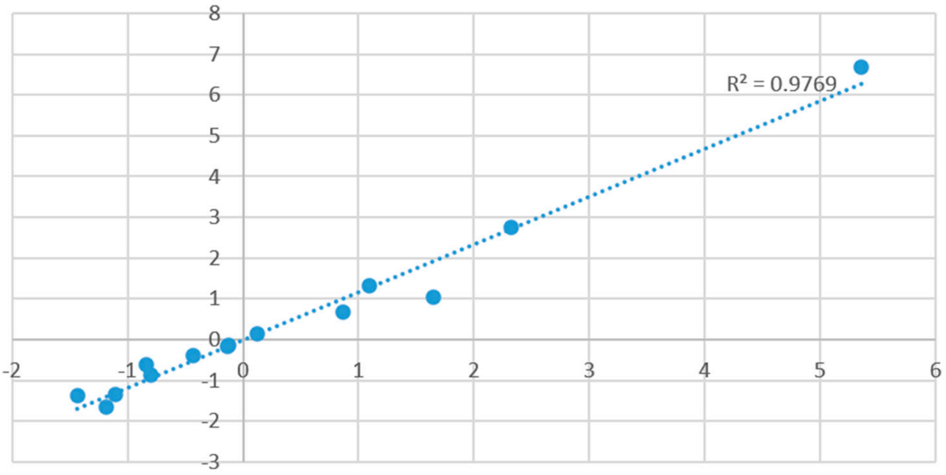

Supplementary Figure S4. Correlation analysis between RNA-seq and qRT-PCR.
