# Supplementary material for: Whole-Transcriptome Analysis Reveals Potential CeRNA Regulatory Mechanism in Takifugu rubripes against Cryptocaryon irritans Infection
Source: Biology (Basel). 2024 Oct 1;13(10):788. doi: 10.3390/biology13100788 (PMC11504436; doi:10.3390/biology13100788)
Supplement: Supplementary file 1 [file biology-13-00788-s001.zip › Supplementary methods and materials.pdf]

Supplementary Methods and Materials

- **RNA qualitative and quantitative analysis**

RNA degradation and contamination was monitored on 1% agarose gels.

RNA purity was checked using the NanoPhotometer® spectrophotometer (IMPLEN, CA, USA)

RNA integrity was assessed using the RNA Nano 6000 Assay Kit ofthe AgilentBioanalyzer 2100 system (Agilent Technologies, CA, USA).

- **Library preparation for Small RNA sequencing**

The small RNA libraries were prepared from a total of 2 ug total RNA isolated from each sampleusing NEBNext" Multiplex Small RNA Library Prep Set for lllumina" (NEB, USA.) according tothe manufacturer's instruction, Briefly, purified RNA was mixed with NEB 3' SR Adapter, then theSR RT Primer hybridized to the excess of 3' SR Adapter (that remained free after the 3' ligationreaction) and transformed the single-stranded DNA adapter into a double-stranded DNA moleculeThis step is important to prevent adapter-dimer formation, besides, dsDNAs are not substrates forligation mediated by T4 RNA Ligase I and therefore do not ligate to the 5'SR Adapter in thesubsequent ligation step. 5'ends adapter was ligated to 5'ends of miRNAs, siRNA and piRNAThen first strand cDNA was synthesized using M-MuLV Reverse Transcriptase (RNase H"). PCRamplification was performed using LongAmp Taq 2X Master Mix, SR Primer for lllumina andindex (X) primer. PCR products were purified on a 8% polyacrylamide gel (100V, 80 min). DNA fragments corresponding to 140-160 bp (the length of small RNA plus the 3' and 5' adapters) wererecovered and dissolved in 8 pL elution bufier. At last, library quality was assessed on the AgilentBioanalyzer 2100 system using DNA High Sensitivity Chips.

- **Clustering and sequencing for Small RNA sequencing**

The clustering of the index-coded samples was performed on a cBot Cluster Generation Systemusing TruSeq SR Cluster Kit v3-cBot-HS (lllumina) according to the manufacturer's instructions.After cluster generation, the library preparations were sequenced on a Novaseq 6000 platform and 50 bp single-end reads were generated.

➢ **Library preparation for lncRNA sequencing**

A total of 1 μg total RNA per sample was used as input material for the lncRNA library preparation. Strand-specific libraries were generated using NEBNext® UltraTM RNA Library Prep Kit for Illumina® (NEB, USA) following manufacturer's recommendations and index codes were added to attribute sequences to each sample. Briefly, ribosome RNA was depleted from total RNA. Fragmentation was carried out using divalent cations under elevated temperature in NEBNext First Strand Synthesis Reaction Buffer(5X). First strand cDNA was synthesized using random hexamer primer and M-MuLV Reverse Transcriptase (RNase H-). Second strand cDNA synthesis was subsequently performed using DNA Polymerase I and RNase H with the incorporation of dUTP instead of dTTP. Remaining overhangs were converted into blunt ends via exonuclease/polymerase activities. After adenylation of 3' ends of DNA fragments, NEBNext Adaptor with hairpin loop structure were ligated for hybridization. In order to select cDNA fragments of preferentially 150-200 bp in length, the library fragments were purified with AMPure XP system (Beckman Coulter, Beverly, USA).

Then USER Enzyme (NEB, USA) was used to digest the second strand with size-selected, adaptor-ligated cDNA at 37°C for 15 min followed by 5 min at 95 °C before PCR. Then PCR was performed with Phusion High-Fidelity DNA polymerase, Universal PCR primers and Index (X) Primer. And then, PCR products were purified (AMPure XP system) and library quality was assessed on the Agilent Bioanalyzer 2100 system.

➢ **Clustering and sequencing for lncRNA sequencing**

The clustering of the index-coded samples was performed on a cBot Cluster Generation System using TruSeq PE Cluster Kit v3-cBot-HS (Illumina) according to the manufacturer's instructions. After cluster generation, the library preparations were sequenced on a Novaseq 6000 platform and 150 bp stand-specific paired-end reads were generated.

- **Library preparation for circRNA sequencing**

A total amount of 5 μg RNA per sample was used as input material for the RNA sample preparations. Firstly, ribosomal RNA was removed by Epicentre Ribozero™ rRNA Removal Kit (Epicentre, USA), and rRNA free residue was cleaned up by ethanol precipitation. Subsequently, the linear RNA was digested with 3 U of RNase R (Epicentre, USA) per μg of RNA. The sequencing libraries were generated by NEBNext® Ultra™ Directional RNA Library Prep Kit for Illumina® (NEB, USA) following manufacturer's recommendations. Briefly, fragmentation was carried out using divalent cations under elevated temperature in NEBNext First Strand Synthesis Reaction Buffer(5X). First strand cDNA was synthesized using random hexamer primer and M-MuLV Reverse Transcriptase (RNaseH-). Second strand cDNA synthesis was subsequently performed using DNA Polymerase I and RNase H. In the reaction buffer, dNTPs with dTTP were replaced by dUTP. Remaining overhangs were converted into blunt ends via exonuclease/polymerase activities. After adenylation of 3' ends of DNA fragments, NEBNext Adaptor with hairpin loop structure were ligated to prepare for hybridization. In order to select cDNA fragments of preferentially 250-300 bp in length, the library fragments were purified with AMPure XP system (Beckman Coulter, Beverly, USA). Then 3 μl USER Enzyme (NEB, USA) was used with size-selected, adaptor-ligated cDNA at 37° C for 15 min followed by 5 min at 95°C before PCR. Then PCR was performed with Phusion High-Fidelity DNA polymerase, Universal PCR primers and Index (X) Primer. At last, products were purified (AMPure XP system) and library quality was assessed on the Agilent Bioanalyzer 2100 system.

- **Clustering and sequencing for circRNA sequencing**

The clustering of the index-coded samples was performed on a cBot Cluster Generation System using TruSeq PE Cluster Kit v3-cBot-HS (Illumina) according to the manufacturer's instructions. After cluster generation, the libraries were sequenced on an Illumina platform and 150 bp pairedend reads were generated.

Table.S4 Reference genome mapped information of LncRNA

Sample name	CG1	CG2	CG3	IG1	IG2	IG3
Total reads	104752894	107165878	106728746	103683634	105323158	105637388
Total mapped	92900438 (88.69%)	95831578 (89.42%)	93079119 (87.21%)	90583879 (87.37%)	95342507 (90.52%)	93165811 (88.19%)
Multiple mapped	14553671 (13.89%)	12312472 (11.49%)	11927192 (11.18%)	11968226 (11.54%)	12311621 (11.69%)	12378400 (11.72%)
Uniquely mapped	78346767 (74.79%)	83519106 (77.93%)	81151927 (76.04%)	78615653 (75.82%)	83030886 (78.3%)	80787411 (76.48%)
Read-1	39256647 (37.48%)	41851511 (39.05%)	40675877 (38.11%)	39133554 (37.74%)	41657257 (39.55%)	40604164 (38.44%)
Read-2	39090120 (37.32%)	41667595 (38.88%)	40476050 (37.92%)	39482099 (38.08%)	41373629 (39.28%)	40183247 (38.04%)
Reads map to +	39159216 (37.38%)	41741543 (38.95%)	40560072 (38.00%)	39241266 (37.85%)	41496252 (39.4%)	40423629 (38.27%)
Reads map to -	39187551 (37.41%)	41777563 (38.98%)	40591855 (38.03%)	39374387 (37.98%)	41534634 (39.44%)	40363782 (38.21%)
Non-splice reads	43382047 (41.41%)	45437589 (42.4%)	44485989 (41.68%)	42452423 (40.94%)	43640406 (41.43%)	45716717 (43.28%)
Splice reads	34964720 (33.20%)	38081517 (35.54%)	36665938 (34.35%)	36163230 (34.88%)	39390480 (37.4%)	35070695 (33.2%)
Reads mapped in proper pairs	75563328 (72.13%)	81096286 (75.67%)	78535358 (73.58%)	75516364 (72.83%)	80101968 (76.05%)	77910898 (73.75%)

Table.S5 Reference genome mapped information of miRNA

Sample	Toatal sRNA	Mapped sRNA	+Mapped sRNA	-Mapped sRNA
**CG1**	12910743 (100%)	12633850 (97.86%)	7133576 (55.25%)	5500274 (42.60%)
**CG2**	14386341 (100%)	13777594 (95.77%)	7750279 (53.87%)	6027315 (41.90%)
**CG3**	14994467 (100%)	14333109 (95.59%)	7272093 (48.50%)	7061016 (47.09%)
**IG1**	15032669 (100%)	14478018 (96.31%)	7023251 (46.72%)	7454767 (49.59)
**IG2**	14162243 (100%)	13862941 (97.89%)	6584628 (46.49%)	7278315 (51.39%)
**IG3**	15150054 (100%)	14323989 (94.55%)	8020883 (52.94%)	6303106 (41.60%)
